# Increased risk of fragility fractures in patients with primary biliary cholangitis

**DOI:** 10.1093/jbmrpl/ziae056

**Published:** 2024-04-19

**Authors:** Jihye Lim, Ye-Jee Kim, Sehee Kim, Jonggi Choi

**Affiliations:** Division of Gastroenterology and Hepatology, Department of Internal Medicine, Yeouido St. Mary’s Hospital, College of Medicine, The Catholic University of Korea, Seoul, 07345, Republic of Korea; Department of Clinical Epidemiology and Biostatistics, Asan Medical Center, University of Ulsan College of Medicine, Seoul, 05505, Republic of Korea; Department of Clinical Epidemiology and Biostatistics, Asan Medical Center, University of Ulsan College of Medicine, Seoul, 05505, Republic of Korea; Department of Gastroenterology, Liver Center, Asan Medical Center, University of Ulsan College of Medicine, Seoul, 05505, Republic of Korea

**Keywords:** cholestasis, hepatic osteodystrophy, primary biliary cholangitis, osteoporosis, fragility fractures

## Abstract

Large-scale studies on the risk of fragility fractures in patients with primary biliary cholangitis (PBC) are limited due to low incidence. We aimed to investigate whether PBC is associated with fragility fractures using real-world nationwide data. The Korean National Health Insurance Service claims data from 2007 to 2020 were analyzed in this population-based cohort study. Patients with PBC (*n* = 4951) were matched with controls (*n* = 19 793) using a 1:4 ratio based on age, sex, and follow-up duration. The primary outcome was fragility fracture, which comprised fractures of the vertebra, hip, distal radius, and proximal humerus. The incidence rates (IRs) and hazard ratios (HRs) were determined to assess the impact of PBC on fragility fractures. During the median follow-up period of 5.37 years, 524 patients in the PBC group had fragility fractures (IR, 18.59/1000 person-years [PYs]). After adjusting for covariates, PBC increased the risk of fragility fractures by 1.63-fold (95% confidence interval, 1.20–2.22; *P* = .002). The vertebra and hip were particularly susceptible to fracture in patients with PBC, with adjusted HRs of 1.77 and 2.23, respectively. In the subgroup analysis, the risk of fragility fracture was 2.53-fold higher in men and 1.59-fold higher in women with PBC than that in the respective matched control groups. Considering the morbidity and mortality related to fragility fractures, increasing awareness of fragility fracture risk and implementing appropriate preventive measures in patients with PBC are imperative.

## Graphical Abstract

**Graphical Abstract:**
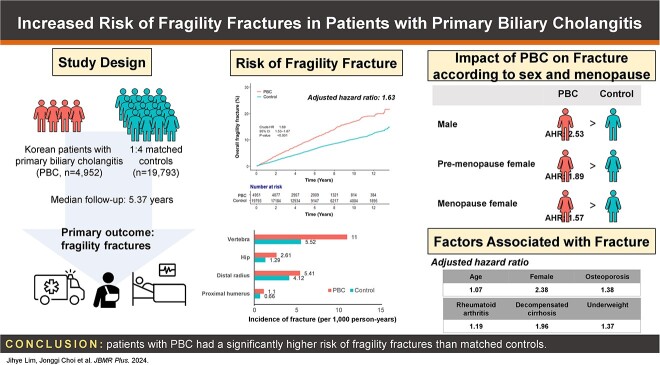

## Introduction

Primary biliary cholangitis (PBC) is a chronic and progressive autoimmune liver disease characterized by nonsuppurative, granulomatous cholangitis that primarily affects the interlobular and septal bile ducts.^1^^,^^2^ If left untreated, PBC can lead to end-stage liver disease, such as liver cirrhosis and hepatic dysfunction. In addition to hepatic complications, patients with PBC often experience extrahepatic manifestations, including fatigue, pruritus, other autoimmune diseases, and osteoporosis.

Hepatic osteodystrophy is a widely recognized condition in which cirrhosis with poor liver function alters bone metabolism and leads to increase osteoporosis and osteoporotic fractures.^3^^,^^4^ Further, PBC may affect bone metabolism through various intricate and multifactorial mechanisms, making patients with PBC particularly vulnerable to osteoporosis.^5^ Cholestasis disturbs the absorption of vitamin D and calcium, which are essential elements for bone mineralization.^5^ Also, elevated bilirubin and consistent inflammation inhibit osteoblast activity.^5^ Indeed, PBC deteriorates bone microstructure, especially by reducing cortical thickening, which is responsible for bone strength.^6^^,^^7^

Osteoporosis and related fractures are well-recognized issues. Osteoporotic fracture increases the incidence of subsequent fractures, morbidity, and mortality, deteriorating quality of life.^8^ Attempts to characterize the relationship between PBC and osteoporotic fractures have been inconclusive and inconsistent.^9^^,^^10^ Because the prevalence of PBC is increasing, large-scale cohort studies are needed to assess the incidence of and risk factors for fragility fractures in patients with PBC.^11^ Therefore, this study aimed to determine the impact of PBC on fragility fractures using Korean nationwide cohort data.

## Material and methods

### Study design and population

This population-based cohort study was conducted using nationwide health insurance claims data from the Korea National Health Insurance Service (NHIS). Korea has a universal health coverage system that provides insurance to almost the entire Korean population. The NHIS manages the national health insurance information database, which stores demographic data, medical records based on the International Classification of Diseases, 10th Revision (ICD-10) codes, and details of drug prescriptions, health check-ups, and use of healthcare facilities.^12^ Patients with rare and incurable diseases (RIDs) are registered as special estimate cases. The NHIS supports 90% of these individuals’ medical expenses and provides them access to new medical technologies and drugs. Classification as a special estimate case requires a qualified physician’s diagnosis based on serologic, imaging, and histologic results.^13^ For example, patients with at least two of the following conditions could be diagnosed with PBC: (1) cholestasis with alkaline phosphatase elevation without evidence of other causes; (2) presence of antimitochondrial antibody or PBC-specific antinuclear antibody immunofluorescence; and (3) characteristic pathology with chronic, nonsuppurative cholangitis.^1^^,^^2^ Based on other previous studies, the accuracy of RIDs in Korea is reliable with the accuracy exceeding 90%.^14^^,^^15^

We included patients newly diagnosed with PBC between 2007 and 2020. PBC diagnosis was identified using two criteria, namely the ICD-10 code for PBC (K74.3) and RID registration code (V174). We excluded the following individuals: (1) 18 patients with insufficient medical information; (2) 603 patients with other chronic liver diseases, such as chronic hepatitis B virus infection, alpha-1 antitrypsin deficiency, hemochromatosis, or Wilson disease; (3) 56 patients with malignancies; (4) 4 patients who received organ transplantation, including liver; and (2) 452 patients with a history of fragility fracture. In total, 4951 patients were selected for the study. Patients with PBC received treatment with ursodeoxycholic acid (UDCA), consistent with the established guidelines.^1^^,^^2^

**Figure 1:**
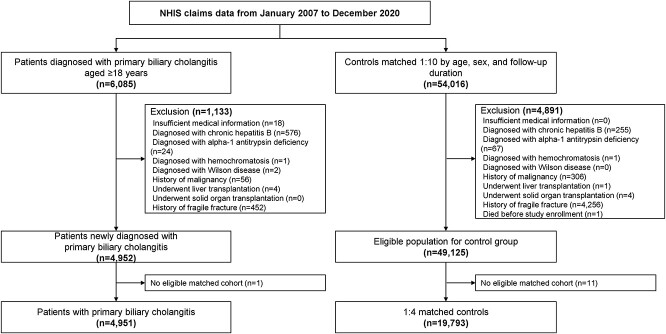
Study flowchart of patient and control selection.

We employed a 1:4 case-to-control ratio to enhance statistical power and ensure robust comparability between patients with PBC and matched controls (19 973 individuals). Controls were meticulously matched to cases based on age, sex, and follow-up duration with additional adjustments made for comorbidities and medication use to accurately assess and compare the risk of fragility fractures. This approach was designed to ensure a balanced comparison, accurately reflecting the health status and minimizing potential bias in fracture risk estimation (Figure 1). This study was conducted in accordance with the Declarations of Helsinki and Istanbul. The Institutional Review Board of Asan Medical Center approved this study (IRB No. 2021-1445), and the requirement for obtaining informed consent was waived as the data were de-identified.

### Data collection

We gathered information on age, sex, socioeconomic status, death, comorbidities, treatment, prescriptions, and health check-ups. Socioeconomic status was based on household income, which was derived from the income-based contribution imposition system; individuals unable to pay insurance contributions are granted support by the medical aid system. Comorbidities claimed in the year prior to PBC diagnosis were analyzed, including hypertension, diabetes mellitus, dyslipidemia, stroke, chronic kidney disease, osteoporosis, anemia, and rheumatoid arthritis. Patients with decompensated cirrhosis were identified as those requiring treatment for ascites (paracentesis or spironolactone), variceal bleeding (endoscopic treatment, terlipressin, or somatostatin), or hepatic encephalopathy (Table S1). Medication use was defined as prescription for at least 30 consecutive days within the first 6 months of PBC diagnosis, while long-term medication use was classified as prescription for more than180 days within the first 2 years of PBC diagnosis. We assessed the use of fibrates (bezafibrate and fenofibrate), glucocorticoids (prednisone, prednisolone, methylprednisolone, deflazacort, betamethasone, dexamethasone, and hydrocortisone), and non-glucocorticoid immunosuppressive agents (azathioprine, mercaptopurine, and mycophenolate mofetil).

The NHIS provides general health check-ups to people aged ≥20 years once every 2 years for early diagnosis of diseases. This includes the collection of anthropometric data; blood pressure measurement; chest X-ray; blood testing including hemoglobin, fasting glucose, aminotransferases, r-glutamyl transpeptidase, and serum creatinine levels; urine testing; visual and hearing assessment; dental examination; and assessment of lifestyle factors, such as alcohol consumption, smoking, and physical activity.^16^ In 2020, 67.8% of all eligible Koreans underwent a health check-up.^17^

### Outcomes

The primary outcome of the study was any fragility fracture, which comprised fractures of the vertebra, hip, distal radius, and proximal humerus.^18^^,^^19^ Overall fractures were defined on an individual basis, capturing the first occurrence of a fragility fracture, regardless of the site. Site-specific fracture outcome was computed per lesion. For the fractures occurring at the same sites, only the initial episode was recorded.

The fragility fractures were validated by operational definitions using ICD-10 codes, procedure codes, radiographic study codes, conservative codes, and hospitalization.^18^^,^^20^ Our study employs an operational definition for fragility fractures that has been validated in numerous prior studies.^18^^,^^20^^,^^21^  Table S2 details the specific definitions and codes utilized. In summary, a diagnosis of a fragility fracture was considered if a fracture code was accompanied by a procedural code (e.g., vertebroplasty or fracture reduction) or if a fracture code was accompanied with hospital admission and relevant radiographic codes.

### Statistical analyses

We included all eligible individuals in the analyses. The matched control group was created using the greedy method, based on age (±2 years), sex, follow-up duration (0–1 year), and health check-up (yes/no). For the PBC group, the index date was defined as the date of RID registration for PBC, and the corresponding matched date was assigned for the control group. The follow-up duration was calculated from the index date to the event of fragility fracture or death, or the date of the last follow-up, December 31, 2020, the date of last follow-up, whichever came first. Conditional logistic regression analysis was used to compare the baseline characteristics of the PBC group and matched control.

We calculated the incidence rate (IR) of fragility fractures per 1000 person-years (PYs) and estimated 95% confidence intervals (CIs) using the Poisson distribution. Thereafter, we used Kaplan–Meier curves to present the cumulative incidence of fragility fractures. Multivariable Cox regression analysis with robust standard error was employed to compare the incidence of fragility fractures between the PBC and control groups. To minimize potential bias, we performed univariate and multivariable analyses, adjusting for following covariables: PBC, age, sex, socioeconomic status, comorbidities, decompensated cirrhosis, body mass index (BMI), smoking, alcohol consumption, exercise, health check-up, and medication use. Multivariable Cox regression was conducted using variables with *P* < .1 in the univariate analysis to identify the risk factors for fragility fractures in patients with PBC. When assessing the risk of overall fracture, we recorded the first fracture event per individual, regardless of the site. For site-specific fractures, each fracture occurring in a different site was considered as a separate event. This methodology was applied consistently in both the Kaplan–Meier curves and Cox regression analysis. Furthermore, to evaluate the influence of PBC on fragility fractures stratified by sex, we conducted a subgroup analysis and calculated adjusted hazard ratios (AHRs) for each sex. We also categorized women into pre- (≤50 years old) and post-menopausal (>50 years old) groups to determine the fracture risk before and after menopause.^22^ Statistical significance was set at *P* < .05, and all statistical analyses were performed using SAS Enterprise Guide version 7.1 (SAS Institute, Inc., Cary, NC, USA).

## Results

### Baseline characteristics

Table 1 presents the demographic and clinical features of the study population. The mean age of patients with PBC was 57.3 years, and 4059 (82.0%) were women. Compared with the matched control group, the PBC group had a higher prevalence of all comorbidities, with higher Charlson Comorbidity Index scores. Decompensated cirrhosis was present in 7.7% of patients in the PBC group but only in 0.2% of individuals in the control (*P* < .001). Additionally, medication use was more frequent in the PBC than in the matched control. Specifically, 93.9% of patients with PBC were treated with UDCAs. The use of glucocorticoids and non-glucocorticoid immunosuppressive agents was also significantly higher in the PBC group than matched controls (12.9% vs. 2.4% and 7.0% vs. 0.1%, respectively; both *P* < .001). In the entire study population, 75.6% of individuals underwent a health check-up. The proportion of patients in the PBC group who consumed alcohol was 14.4%, which was significantly different from the 22.2% in the non-PBC group. In contrast, no statistically significant differences in smoking or exercise habits were identified between the two groups.

**Table 1:** Baseline characteristics of patients with primary biliary cholangitis and matched controls.

	PBC (*n* = 4951)	Matched controls (*n* = 19 793)	*P*-value
Follow-up duration, median (IQR), y	5.37 [2.88, 8.86]	5.96 [3.28, 9.59]	<.001
Age, mean (SD), y	57.3 (11.9)	57.4 (11.8)	.675
Age, *n* (%)			.743
18–39 years	348 (7.0)	1331 (6.7)	
40–64 years	3237 (65.4)	12 966 (65.5)	
≥65 years	1366 (27.6)	5496 (27.8)	
Female sex, *n* (%)	4059 (82.0)	16 236 (82.0)	.940
Socioeconomic status, *n* (%)			<.001
National health insurance	4613 (93.2)	18 482 (93.4)	
Household income <30%	950 (19.2)	4552 (23.0)	
Household income 30–70%	1506 (30.4)	6452 (32.6)	
Household income ≥70%	2157 (43.6)	7478 (37.8)	
Medical aid	244 (4.9)	818 (4.1)	
Unknown	94 (1.9)	493 (2.5)	
Comorbidities, *n* (%)			
Hypertension	1708 (34.5)	6299 (31.8)	<.001
Diabetes mellitus	1547 (31.2)	3612 (18.2)	<.001
Dyslipidemia	3235 (65.3)	6297 (31.8)	<.001
Stroke	207 (4.2)	686 (3.5)	.016
Chronic kidney disease	141 (2.8)	247 (1.2)	<.001
Osteoporosis	978 (19.8)	2528 (12.8)	<.001
Anemia	1059 (21.4)	1059 (5.4)	<.001
Rheumatoid arthritis	599 (12.1)	788 (4.0)	<.001
Hypogonadism	17 (0.3)	21 (0.1)	<.001
CCI score, mean (SD)	3.3 (2.4)	1.4 (1.9)	<.001
CCI score, *n* (%)			<.001
0	247 (5.0)	8356 (42.2)	
1	966 (19.5)	4758 (24.0)	
2	976 (19.7)	2797 (14.1)	
≥3	2762 (55.8)	3882 (19.6)	
Diagnosis period, *n* (%)			.359
2007-2010	1174 (23.7)	4504 (22.8)	
2011-2015	1770 (35.8)	7166 (36.2)	
2016-2019	2007 (40.5)	8123 (41.0)	
Decompensated cirrhosis, *n* (%)	383 (7.7)	35 (0.2)	<.001
Medication use^a^, *n* (%)			
Ursodeoxycholic acid	4650 (93.9)	266 (1.3)	<.001
Fibrate	50 (1.0)	233 (1.2)	.32
Glucocorticoid	641 (12.9)	471 (2.4)	<.001
Non-glucocorticoid immunosuppressive agent^b^	348 (7.0)	20 (0.1)	<.001
Available for health check-up^c^	3744 (75.6)	14 967 (75.6)	.996
Body mass index, mean (SD), kg/m^2^	23.3 (3.1)	23.9 (3.3)	<.001
Body mass index, *n* (%)			<.001
<18.5 kg/m^2^	154 (3.1)	430 (2.2)	
18.5–22.9 kg/m^2^	1649 (33.3)	5726 (28.9)	
23.0–24.9 kg/m^2^	958 (19.3)	3732 (18.9)	
≥25 kg/m^2^	983 (19.9)	5079 (25.7)	
Unknown	1207 (24.4)	4826 (24.4)	
Smoking			.799
Never or past	3098 (62.6)	12 477 (63.0)	
Current	589 (11.9)	2300 (11.6)	
Unknown	1264 (25.5)	5016 (25.3)	
Alcohol consumption			<.001
Non-drinker	2977 (60.1)	10 389 (52.5)	
1–4 times per week	670 (13.5)	4048 (20.5)	
≥5 times per week	43 (0.9)	337 (1.7)	
Unknown	1261 (25.5)	5019 (25.4)	
Exercise at least once per week			.288
No	1781 (36.0)	7351 (37.1)	
Yes	1904 (38.5)	7423 (37.5)	
Unknown	1266 (25.6)	5019 (25.4)	

Abbreviation: CCI, Charlson Comorbidity Index; IQR, interquartile range; PBC, primary biliary cholangitis; SD, standard deviation

a Medication use was defined as prescription of the relevant drug for ≥30 consecutive days within the first 6 months of PBC diagnosis.

b Non-glucocorticoid immunosuppressive agents include azathioprine, mercaptopurine, and mycophenolate mofetil.

c The data for body mass index, smoking, alcohol consumption, and exercise at least once per week were available for participants who underwent health check-up.

### Fragility fractures

**Figure 2:**
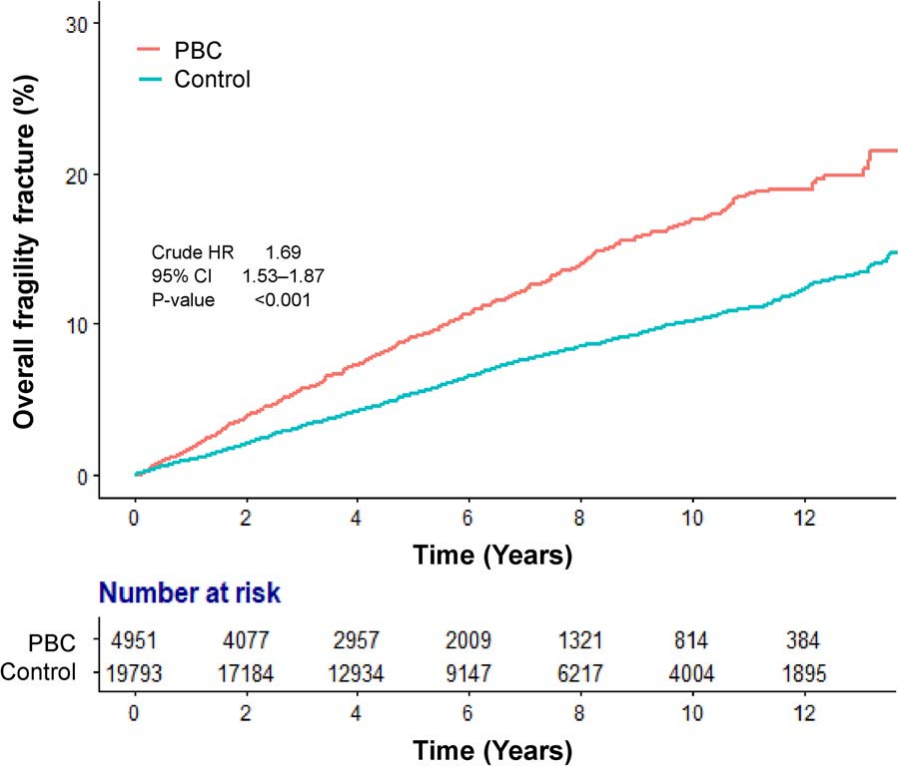
Cumulative risk of fragility fractures in patients with primary biliary cholangitis and the matched control group.

The median follow-up duration for patients with PBC was 5.37 years (interquartile range [IQR], 2.88–8.86 years). A total of 524 fragility fractures was observed in the PBC group, resulting in an IR of 18.59/1000 PYs (95% CI, 17.03–20.25), as shown in Table 2. Out of the 4951 patients with PBC, 524 experienced a fracture during the study period; 462 patients experienced a fracture at only one site, and 62 patients experienced fractures at 2 or more sites. Among the 19 793 matched controls, 1355 individuals experienced a fracture; 1246 patients had only one site fracture, while 109 patients had fractures at 2 or more sites. The PBC group had a higher risk of overall fragility fracture than the matched control, with a crude HR of 1.69 (95% CI, 1.53–1.87). The cumulative risk of fragility fracture for patients with PBC was 3.9%, 9.2%, 12.3%, and 16.9% at 2, 5, 7, and 10 years, respectively. By comparison, the matched control had fragility fracture rates of 2.1%, 5.4%, 7.6%, and 10.2% at the corresponding times, as illustrated in Figure 2. PBC was independently associated with the fragility fractures after adjusting for associated covariates, with an AHR of 1.63 (95% CI, 1.20–2.22; Table 3). In addition, older age, female sex, osteoporosis, rheumatoid arthritis, decompensated cirrhosis, and underweight (BMI < 18.5 kg/m^2^) increased the risk of fragility fractures.

**Table 2:** Observed cases and incidence rates of fragility fractures.

	PBC			Matched control cohort		
	Observed cases	Sum of PYs	Incidence rate^a^ (95% CI)	Observed cases	Sum of PYs	Incidence rate^a^ (95% CI)
Overall fracture	524	28185.7	18.59 (17.03–20.25)	1355	123365.5	10.98 (10.41–11.58)
Vertebral fracture	319	29011.9	11.00 (9.82–12.27)	698	126495.1	5.52 (5.12–5.94)
Hip fracture	78	29896.0	2.61 (2.06–3.26)	166	128973.5	1.29 (1.10–1.50)
Distal radius fracture	159	29398.2	5.41 (4.60–6.32)	524	127167.2	4.12 (3.78–4.49)
Proximal humerus fracture	33	29977.9	1.10 (0.76–1.55)	85	129316.0	0.66 (0.53–0.81)

Abbreviation: CI, confidence interval; PBC, primary biliary cholangitis; PY, person-years.

a Incidence rate per 1000 PYs.

**Table 3:** Multivariable Cox regression analysis of overall fragility fracture.

	Model 1^a^		Model 2^b^		Model 3^c^	
	HR (95% CI)	*P*-value	HR (95% CI)	*P*-value	HR (95% CI)	*P*-value
Primary biliary cholangitis	1.65 (1.48–1.85)	<.001	1.66 (1.48–1.85)	<.001	1.63 (1.20–2.22)	.002
Age	1.07 (1.06–1.07)	<.001	1.07 (1.06–1.07)	<.001	1.07 (1.06–1.07)	<.001
Sex						
Male	Reference		Reference		Reference	
Female	2.36 (2.02–2.76)	<.001	2.37 (1.98–2.83)	<.001	2.38 (1.99–2.85)	<.001
Socioeconomic status						
Income <30%	Reference		Reference		Reference	
Income 30–70%	0.88 (0.72–1.07)	.199	0.89 (0.72–1.09)	.261	0.89 (0.73–1.10)	.281
Income ≥70%	0.88 (0.73–1.07)	.195	0.89 (0.73–1.09)	.253	0.89 (0.73–1.09)	.266
Medical aid	0.80 (0.66–0.96)	.016	0.81 (0.67–0.98)	.033	0.81 (0.67–0.99)	.036
Unknown	0.72 (0.49–1.06)	.094	0.73 (0.50–1.08)	.113	0.74 (0.50–1.08)	.118
Comorbidity						
Hypertension	1.05 (0.95–1.17)	.35	1.05 (0.94–1.17)	.354	1.05 (0.94–1.17)	.385
Diabetes mellitus	1.03 (0.92–1.15)	.624	1.02 (0.92–1.15)	.662	1.02 (0.92–1.15)	.662
Dyslipidemia	0.94 (0.85–1.04)	.247	0.95 (0.85–1.05)	.3	0.94 (0.85–1.05)	.299
Stroke	1.09 (0.89–1.32)	.405	1.08 (0.89–1.32)	.417	1.09 (0.90–1.33)	.393
Chronic kidney disease	1.06 (0.77–1.46)	.722	1.06 (0.77–1.46)	.712	1.06 (0.77–1.46)	.741
Osteoporosis	1.38 (1.24–1.54)	<.001	1.38 (1.24–1.55)	<.001	1.38 (1.23–1.54)	<.001
Anemia	1.13 (0.98–1.32)	.097	1.12 (0.97–1.30)	.124	1.12 (0.96–1.30)	.141
Rheumatoid arthritis	1.22 (1.03–1.45)	.021	1.22 (1.02–1.44)	.025	1.19 (1.00–1.42)	.045
Hypogonadism	2.31 (0.88–6.11)	.091	2.18 (0.82–5.81)	.118	2.08 (0.78–5.54)	.141
Decompensated cirrhosis	2.01 (1.55–2.59)	<.001	1.99 (1.54–2.56)	<.001	1.96 (1.51–2.53)	<.001
Body mass index						
18.5–22.9 kg/m^2^			Reference		Reference	
<18.5 kg/m^2^			1.37 (1.04–1.81)	.025	1.37 (1.04–1.81)	.024
23.0–24.9 kg/m^2^			0.89 (0.78–1.02)	.1	0.89 (0.77–1.02)	.097
≥25 kg/m^2^			1.04 (0.92–1.18)	.552	1.04 (0.91–1.18)	.556
Smoking						
Never or past			Reference		Reference	
Current			1.07 (0.86–1.33)	.544	1.07 (0.86–1.33)	.548
Alcohol consumption						
Non-drinker			Reference		Reference	
1–4 times per week			0.87 (0.73–1.03)	.114	0.87 (0.73–1.04)	.119
≥5 times per week			1.16 (0.74–1.83)	.521	1.16 (0.74–1.83)	.514
Exercise at least once per week						
No			Reference		Reference	
Yes			0.90 (0.80–1.00)	.051	0.90 (0.80–1.00)	.052
Health check-up			1.04 (0.90–1.20)	.581	1.04 (0.91–1.20)	.574
Medication use^d^						
Ursodeoxycholic acid					0.99 (0.73–1.35)	.967
Fibrate					1.04 (0.68–1.59)	.862
Glucocorticoid					1.18 (0.95–1.48)	.132
Non-glucocorticoid immunosuppressive agent^e^					1.18 (0.82–1.69)	.383

Abbreviation: CI, confidence interval; HR, hazard ratio.

a Model 1: adjusted for age, sex, socioeconomic status, comorbidities, and decompensated cirrhosis.

b Model 2: adjusted for age, sex, socioeconomic status, comorbidities, decompensated cirrhosis, body mass index, smoking, alcohol, exercise, and health check-up.

c Model 3: adjusted for age, sex, socioeconomic status, comorbidities, decompensated cirrhosis, body mass index, smoking, alcohol, exercise, health check-up, and medication use.

d Medication use was defined as prescription of the relevant drug for ≥30 days within the first 6 months of PBC diagnosis.

e Non-glucocorticoid immunosuppressive agents include azathioprine, mercaptopurine, and mycophenolate mofetil.

**Figure 3:**
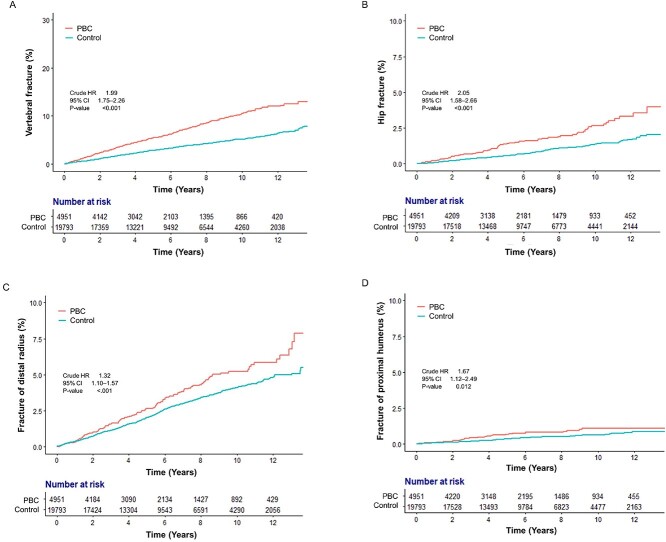
Cumulative risks of site-specific fragility fractures in patients with primary biliary cholangitis and the matched control group. (A) Vertebral fracture. (B) Hip fracture. (C) Distal radius fracture. (D) Proximal humerus fracture.

In the PBC cohort, the vertebra was the most commonly fractured site, with an IR of 11.00/1000 PYs (95% CI, 9.82–12.27), followed by the distal radius (IR, 5.41/1000 PYs; 95% CI, 4.60–6.32), hip (IR, 2.61/1000 PYs; 95% CI, 2.06–3.26), and proximal humerus (IR, 1.10/1000 PYs; 95% CI, 0.76–1.55). The order of fracture site incidence was the same in the matched control, although each IR was lower than that in the PBC group. Among the fracture sites, the risk of hip fracture was the highest before (crude HR, 2.05; 95% CI, 1.58–2.66) and after adjustment (AHR, 2.23; 95% CI, 1.04–4.76; Figure 3 and Table S3). Vertebral fracture risk was also high, with an AHR of 1.77 (95% CI, 1.18–2.65). When adjusted, the risk of distal radius (AHR, 1.39; 95% CI, 0.71–2.73) and proximal humerus (AHR, 1.27; 95% CI, 0.44–3.70) fractures was also increased in the PBC group; however, the results were not statistically significant (Table S3).

### Subgroup analysis: 2-year landmark analysis

Of the 4951 patients with PBC, 4077 who were followed up for ≥2 years were included in the 2-year landmark analysis to minimize interference from incidental fractures that occurred independently of PBC. Table S4 presents the characteristics of these patients. The median follow-up duration was 6.32 years (IQR, 4.01–9.60 years). The analysis revealed a significantly increased risk of overall fragility fracture in patients with PBC (AHR, 1.77; 95% CI, 1.29–2.42; Table S5). The risk of vertebral fracture was also higher in patients with PBC compared to that in matched controls (AHR, 1.82; 95% CI, 1.18–2.81;

Table S6). Additionally, the risks of hip and proximal humerus fractures tended to be higher in patients with PBC than matched controls, although this difference did not reach statistical significance (Table S6).

### Subgroup analysis: impact of PBC on fragility fractures according to sex and menopause

Of the 24 744 individuals, 4449 (18.0%) were men. The overall fragility fracture risk AHR for men with PBC was 2.53 (95% CI, 1.84–3.48). Women with PBC had a 1.59-fold higher risk of overall fragility fracture (95% CI, 1.41–1.79) compared to matched female controls (Table S7). Nevertheless, the incidence rate, reflecting the disease burden from fragility fractures, was higher among women than men. Additionally, postmenopausal women showed a higher incidence of fragility fractures compared to premenopausal women (Table S8).

## Discussion

This nationwide population-based cohort study revealed a 63% increased risk of fragility fractures associated with PBC. Patients with PBC had a 1.77- and 2.23-fold greater risk of vertebral and hip fractures, respectively, compared with matched controls. Furthermore, PBC had a greater influence on fragility fractures in men (AHR, 2.53) than in women (AHR, 1.59).

Previous studies have suggested an association between PBC and an increased risk of osteoporotic fracture.^9^^,^^10^ A study in the United Kingdom, which included 930 patients with PBC and 9202 matched controls, showed that patients with PBC had an approximate 2-fold increased risk of osteoporotic fractures compared to the controls.^9^ However, the study included patients with other chronic liver diseases, such as autoimmune hepatitis and primary sclerosing cholangitis; further, 48.7% of the patients with PBC did not receive a UDCA prescription, while 29.5% were prescribed corticosteroids. A subsequent Swedish study that assessed 3980 patients with PBC and 37 196 matched controls showed that PBC increased the risk of osteoporotic fractures by 1.6-fold over the 4.3-year follow-up.^10^ However, this finding might be inconclusive because more patients in the PBC population had other risk factors for osteoporotic fractures than those in the control group at enrollment, some of which were not adjusted for; these included malignancies, osteoporosis, and previous fractures.^19^^,^^23^ Considering the low incidence of PBC and fragility fractures, we included a larger dataset collected over a longer period and considered various other factors relevant to fragility fracture. Therefore, we believe our findings provide more definitive evidence on the risk of fractures in patients with PBC. Possible explanations for bone vulnerability in patients with PBC may originate from characteristic features of the disease itself. Cumulative injury to biliary epithelial cells leads to cholestasis, impairing the absorption of fat-soluble vitamins, essential for bone formation.^24^ Additionally, elevated bilirubin and bile acids inhibit osteoblastic activity. Increased levels of inflammatory cytokines further promote osteoclast activity, contributing to bone resorption.^5^

In our study, vertebral and hip fractures were the most common and vulnerable fracture sites in patients with PBC. The vertebra and hip are weight-bearing bones that cause serious mobility impairment when fractured. This, in turn, may lead to conditions, such as deep vein thrombosis, bedsores, or pneumonia.^25^ The 1-year mortality rate after weight-bearing bone fractures is upto 24.2%.^26^ The mortality rate after osteoporotic fractures is higher in patients with PBC than in those without PBC.^3^^,^^10^ This may be explained by chronic hepatic inflammation, high disease burden, and other comorbidities observed in patients with PBC, which interfere with the wound healing process and increase the risk of complications.^27^

The impact of PBC on fragility fractures was 2.53 times higher in men compared to 1.59 times higher in women when compared to their matched controls, respectively. Additionally, having PBC increased the risk of fragility fractures by 1.89-fold in premenopausal women and 1.57-fold in postmenopausal women. Nevertheless, the incidence rate, reflecting the disease burden from fragility fractures, was higher among women than men. Additionally, postmenopausal women showed a higher incidence of fragility fractures compared to premenopausal women (Table S8). Therefore, factors other than estrogen levels may have contributed to the increased vulnerability of men with PBC to osteoporosis and related fractures in our study. Because of its lower prevalence in men, osteoporosis is often overlooked and remains underdiagnosed and undertreated in men.^28^ However, approximately 30% of older men have osteoporosis, and bone loss is accelerated in chronic diseases such as PBC.^28^ Furthermore, men have a higher fracture-related mortality than women. The importance of the observed increased risk of fragility fractures in men with PBC should not be underestimated, and additional research is needed to elucidate its underlying mechanisms.

Efforts have been made to prevent osteoporosis in patients with PBC, including control of PBC itself. UDCA has survival benefits in patients with PBC, although its efficacy in preventing osteoporosis is yet to be established.^29^ UDCA increases osteoblast differentiation and activity at the cellular level.^30^ However, the drug had no effect on osteoporotic fracture rates in previous population-based studies.^9^^,^^31^ Similarly, UDCA and other medications had no influence on fragility fracture incidence in our study. Further comprehensive investigation is required to confirm the role of UDCA in bone metabolism in patients with PBC.

We conducted a nationwide population-based cohort study spanning 14 years. However, the study had several limitations. First, due to the innate limitations of the claims data, we were unable to obtain information on liver function or osteoporosis indicators, such as liver biochemical test results or bone mineral density. Although we defined osteoporosis using ICD-10 codes, the actual prevalence of osteoporosis in patients with PBC is unknown. The lack of liver biochemical tests also made it challenging to assess the relationship between liver function and fragility fractures. However, by defining decompensated cirrhosis using procedure codes and prescriptions, we aimed to gain insights into the association between the severity of hepatic dysfunction and fragility fractures. Second, patients who experienced fragility fractures before PBC diagnosis were excluded from the study, which might have underestimated the risk of fragility fracture, as the first fragility fracture increases the risk of subsequent fractures. Furthermore, the attributable risk of fragility fractures directly associated with PBC could not be accurately determined in our study due to the limitations inherent in its design. Finally, asymptomatic patients with undiagnosed radiographic vertebral fractures, who did not seek medical attention, were not included in our analysis despite having fragility fractures.

In conclusion, this study showed that patients with PBC had a significantly higher risk of fragility fractures than matched controls. Specifically, patients with PBC were more vulnerable to vertebral and hip fractures, which may negatively impact quality of life and life expectancy. Considering the increased risk of fragility fractures in patients with PBC, awareness of fragility fracture risk and establishment of preventive measures is essential.

## Supplementary Material

JBMR_plus_Supp_rev_v2_ziae056

## Data Availability

The data that support the findings of this study are available from the Korea National Health Insurance Service (NHIS). Restrictions apply to the availability of these data, which were used under license for this study.
