# Supplementary material for: Increased risk of fragility fractures in patients with primary biliary cholangitis
Source: JBMR Plus. 2024 Apr 19;8(7):ziae056. doi: 10.1093/jbmrpl/ziae056 (PMC11162592; doi:10.1093/jbmrpl/ziae056)
Supplement: JBMR_plus_Supp_rev_v2_ziae056 [file jbmr_plus_supp_rev_v2_ziae056.docx]

**Supplementary Information**

**Increased Risk of Fragility Fractures
in Patients with Primary Biliary Cholangitis**

Jihye Lim, Ye-Jee Kim, Seonok Kim, and Jonggi Choi

**Table of contents**

- **Supplementary Table 1.** Definition of comorbidities in the present study
- **Supplementary Table 2.** Definition of fragility fractures and diagnostic, procedure, radiographic, and conservative codes used to identify fragility fractures in the present study
- **Supplementary Table 3.** Multivariable Cox regression analysis of site-specific fragility fractures
- **Supplementary Table 4.** Two-year characteristics of patients with primary biliary cholangitis (PBC) and matched controls
- **Supplementary Table 5.** Two-year landmark analysis for multivariable Cox regression analysis of overall fragility fracture
- **Supplementary Table 6.** Two-year landmark analysis for multivariable Cox regression analysis of site-specific fragility fractures
- **Supplementary Table 7.** Risk for overall fragility fractures for patients with PBC, compared with their matched controls
- **Supplementary Table 8.** Overall incidence of fragility fractures stratified by sex and menopausal status

**Supplementary Table 1.** Definition of comorbidities in the present study

| Disease | ICD-10 code | RID  code | Procedure  code | Drug code |
| --- | --- | --- | --- | --- |
| Alpha-1 antitrypsin deficiency | E880 |  |  |  |
| Anemia | D50, D51, D52, D53 |  |  |  |
| Chronic hepatitis B | B18.0, B18.1 |  |  |  |
| Chronic kidney disease | N18, I12.0, E14.2 |  |  |  |
| Decompensated cirrhosis | M74 with management for varix or ascites |  | Endoscopic variceal bleeding control: Q7631, Q7633 | Terlipressin 236001BIJ, 236003BIJ, 236030BIJ Somatostatin 230201BIJ, 230203BIJ, 230202BIJ |
|  |  |  | Paracentesis: C8050 | Spironolactone 231101ATB, 231102ATB |
| Diabetes mellitus | E10, E11, E12, E13, E14 |  |  |  |
| Dyslipidemia | E78 |  |  |  |
| Hereditary haemochromatosis | E83.1 |  |  |  |
| Hypertension | I10, I11, I12, I13, I14, I15 |  |  |  |
| Hypogonadism | E23.0, E29.1, E28.3 |  |  |  |
| Liver transplantation | Z94,4 T86.4 |  |  |  |
| Malignancy | C00–C97 | V193, V194, V027 |  |  |
| Osteoporosis | M80, M81, M82 |  |  |  |
| Primary biliary cholangitis | K74.3 | V174 |  |  |
| Rheumatoid arthritis | M05, M06 |  |  |  |
| Solid organ transplantation | Z94, T86 |  |  |  |
| Stroke | I60, I61, I62, I63, I64 |  |  |  |
| Wilson disease | E83.0 | V119 |  |  |

Abbreviations: ICD-10, International Classification of Diseases, 10th Revision; RID, rare and incurable disease

**Supplementary Table 2.** Definition of fragility fractures and diagnostic, procedure, radiographic, and conservative codes used to identify fragility fractures in the present study

| **Codes** | **Description** |
| --- | --- |
| **Operational definition**  *Vertebral fracture*  *Hip fracture*  *Proximal humerus fracture*  *Distal radius fracture* | ICD-10 code and procedure codes  ICD-10 code and radiographic study codes  ICD-10 code and hospitalization  ICD-10 code, hospitalization, and procedure codes  ER visit and procedure code  ICD-10 code and conservative codes  ICD-10 code and hospitalization  ICD-10 code and more than three outpatient clinic visits  ICD-10 code and procedure codes  ICD-10 code and conservative codes  ICD-10 code and procedure codes |
| Diagnostic codes  *Vertebral fracture*  M48.4  M48.5  M49.5  M80.88  S22.0  S22.1  S32.0  S32.7  T08  *Hip fracture*  S72.0  S72.1  *Humerus fracture*  S42.2  S42.3  *Distal radius fracture*  S52.5  S52.6 | According to ICD-10  Fatigue fracture of the vertebra  Collapsed vertebra, not elsewhere classified  Collapsed vertebra in diseases classified elsewhere  Other osteoporosis with pathologic fracture, other (vertebral column)  Fracture of the thoracic vertebra  Multiple fractures of the thoracic spine  Fracture of the lumbar vertebra  Multiple fractures of the lumbar spine  Fracture of the spine  Fracture of the femur neck  Trochanteric fracture  Fracture of the upper end of the humerus  Fracture of the shaft of the humerus    Fracture of the lower end of the radius  Fracture of the lower end of both the ulnar and radius |
| Procedure codes  *Spine fracture*  *Hip fracture*  *Humerus fracture*  *Distal radius fracture* | N0471, N0472, N0473, N0474, N0630  N0601, N0611, N0641, N0652, N0654, N0711, N0715, N0991,  N0981, N2070, N2710  N2076  N0602, N0612, N0722, N0982, N0986, N0992, N2071, N2076,  N2711, N2716 |
| Radiographic study codes  *Thoracic spine*  *Thoracolumbar spine*  *Lumbar spine*  *Lumbosacral spine* | G430  G440  G450  G460 |
| Conservative codes  *Humerus fracture*  *Distal radius fracture* | T0610, T6110  T6020, T6030, T6151, T6152 |

Abbreviations: ER, emergency room; ICD-10, International Classification of Diseases, 10th Revision

**Supplementary Table 3.** Multivariable Cox regression analysis of site-specific fragility fractures

|  | **Overall fracture** | | **Vertebral fracture** | | **Hip fracture** | | **Distal radius fracture** | | **Proximal humerus fracture** | |
| --- | --- | --- | --- | --- | --- | --- | --- | --- | --- | --- |
|  | HR**^*^** (95% CI) | P- value | HR**^*^** (95% CI) | P- value | HR**^*^** (95% CI) | P- value | HR**^*^** (95% CI) | P- value | HR**^*^** (95% CI) | P- value |
| Primary biliary cholangitis | 1.63 (1.20–2.22) | 0.002 | 1.77 (1.18–2.65) | 0.006 | 2.23 (1.04–4.76) | 0.039 | 1.39 (0.71–2.73) | 0.338 | 1.27 (0.44–3.70) | 0.657 |
| Age | 1.07 (1.06–1.07) | <0.001 | 1.09 (1.08–1.09) | <0.001 | 1.10 (1.08–1.11) | <0.001 | 1.03 (1.03–1.04) | <0.001 | 1.03 (1.02–1.05) | <0.001 |
| Sex |  |  |  |  |  |  |  |  |  |  |
| Male | Reference |  | Reference |  | Reference |  | Reference |  | Reference |  |
| Female | 2.38 (1.99–2.85) | <0.001 | 2.10 (1.67–2.65) | <0.001 | 1.85 (1.21–2.84) | 0.005 | 3.42 (2.35–4.97) | <0.001 | 3.09 (1.24–7.73) | 0.016 |
| Socioeconomic status |  |  |  |  |  |  |  |  |  |  |
| Income <30% | Reference |  | Reference |  | Reference |  | Reference |  | Reference |  |
| Income 30–70% | 0.89 (0.73–1.10) | 0.281 | 0.76 (0.58–0.99) | 0.042 | 0.53 (0.32–0.89) | 0.015 | 1.19 (0.80–1.77) | 0.388 | 1.54 (0.68–3.52) | 0.303 |
| Income ≥70% | 0.89 (0.73–1.09) | 0.266 | 0.84 (0.66–1.08) | 0.183 | 0.68 (0.43–1.08) | 0.103 | 1.05 (0.71–1.55) | 0.793 | 1.27 (0.57–2.87) | 0.557 |
| Medical aid | 0.81 (0.67–0.99) | 0.036 | 0.70 (0.54–0.89) | 0.004 | 0.66 (0.43–1.00) | 0.053 | 1.05 (0.72–1.55) | 0.782 | 1.36 (0.64–2.91) | 0.426 |
| Unknown | 0.74 (0.50–1.08) | 0.118 | 0.79 (0.48–1.30) | 0.359 | 0.50 (0.15–1.63) | 0.248 | 0.49 (0.22–1.13) | 0.094 | 2.41 (0.69–8.41) | 0.169 |
| Comorbidity |  |  |  |  |  |  |  |  |  |  |
| Hypertension | 1.05 (0.94–1.17) | 0.385 | 1.07 (0.92–1.25) | 0.37 | 1.16 (0.85–1.56) | 0.347 | 0.96 (0.79–1.16) | 0.659 | 1.32 (0.90–1.94) | 0.16 |
| Diabetes mellitus | 1.02 (0.92–1.15) | 0.662 | 1.03 (0.89–1.19) | 0.707 | 1.29 (0.96–1.73) | 0.094 | 0.89 (0.73–1.08) | 0.243 | 1.36 (0.86–2.15) | 0.184 |
| Dyslipidemia | 0.94 (0.85–1.05) | 0.299 | 0.89 (0.77–1.03) | 0.12 | 1.07 (0.79–1.45) | 0.649 | 0.97 (0.81–1.17) | 0.786 | 1.32 (0.85–2.06) | 0.214 |
| Stroke | 1.09 (0.90–1.33) | 0.393 | 1.09 (0.85–1.39) | 0.498 | 1.31 (0.85–1.99) | 0.216 | 0.99 (0.68–1.45) | 0.977 | 1.24 (0.62–2.47) | 0.543 |
| Chronic kidney disease | 1.06 (0.77–1.46) | 0.741 | 0.71 (0.45–1.14) | 0.16 | 2.16 (1.24–3.77) | 0.007 | 0.99 (0.52–1.88) | 0.969 | 1.61 (0.62–4.14) | 0.326 |
| Osteoporosis | 1.38 (1.23–1.54) | <0.001 | 1.49 (1.29–1.72) | <0.001 | 1.02 (0.76–1.37) | 0.893 | 1.50 (1.24–1.81) | <0.001 | 1.27 (0.81–1.99) | 0.301 |
| Anemia | 1.12 (0.96–1.30) | 0.141 | 1.10 (0.90–1.33) | 0.352 | 1.40 (0.96–2.04) | 0.076 | 1.11 (0.85–1.45) | 0.43 | 1.14 (0.64–2.02) | 0.666 |
| Rheumatoid arthritis | 1.19 (1.00–1.42) | 0.045 | 1.23 (0.98–1.55) | 0.073 | 0.82 (0.46–1.43) | 0.48 | 1.09 (0.81–1.48) | 0.558 | 1.31 (0.66–2.58) | 0.437 |
| Hypogonadism | 2.08 (0.78–5.54) | 0.141 | 2.01 (0.55–7.32) | 0.288 | 3.66 (0.57–23.47) | 0.172 | 1.33 (0.18–10.02) | 0.783 |  |  |
| Decompensated cirrhosis | 1.96 (1.51–2.53) | <.001 | 1.94 (1.41–2.67) | <.001 | 2.53 (1.41–4.54) | 0.002 | 1.29 (0.73–2.29) | 0.374 | 0.72 (0.17–3.12) | 0.666 |
| Health check-up | 1.04 (0.91–1.20) | 0.574 | 1.06 (0.87–1.29) | 0.565 | 0.65 (0.44–0.98) | 0.039 | 1.39 (1.10–1.76) | 0.005 | 0.54 (0.32–0.93) | 0.027 |
| Body mass index |  |  |  |  |  |  |  |  |  |  |
| 18.5–22.9 kg/m^2^ | Reference |  | Reference |  | Reference |  | Reference |  | Reference |  |
| <18.5 kg/m^2^ | 1.37 (1.04–1.81) | 0.024 | 1.29 (0.88–1.88) | 0.185 | 3.67 (2.11–6.37) | <0.001 | 0.74 (0.42–1.28) | 0.276 | 3.71 (1.54–8.92) | 0.003 |
| 23.0–24.9 kg/m^2^ | 0.89 (0.77–1.02) | 0.097 | 1.01 (0.83–1.22) | 0.945 | 0.92 (0.58–1.46) | 0.721 | 0.75 (0.59–0.94) | 0.013 | 0.76 (0.39–1.50) | 0.435 |
| ≥25 kg/m^2^ | 1.04 (0.91–1.18) | 0.556 | 1.14 (0.95–1.37) | 0.153 | 1.11 (0.74–1.67) | 0.601 | 0.95 (0.78–1.16) | 0.603 | 1.12 (0.63–1.98) | 0.704 |
| Smoking |  |  |  |  |  |  |  |  |  |  |
| Never or past | Reference |  | Reference |  | Reference |  | Reference |  | Reference |  |
| Current | 1.07 (0.86–1.33) | 0.548 | 1.26 (0.95–1.67) | 0.111 | 1.40 (0.81–2.41) | 0.224 | 0.77 (0.50–1.18) | 0.224 | 0.99 (0.37–2.64) | 0.977 |
| Alcohol consumption |  |  |  |  |  |  |  |  |  |  |
| Non-drinker | Reference |  | Reference |  | Reference |  | Reference |  | Reference |  |
| 1–4 times per week | 0.87 (0.73–1.04) | 0.119 | 0.87 (0.68–1.12) | 0.293 | 0.52 (0.26–1.04) | 0.067 | 0.88 (0.68–1.14) | 0.338 | 0.96 (0.45–2.07) | 0.921 |
| ≥5 times per week | 1.16 (0.74–1.83) | 0.514 | 1.03 (0.54–1.94) | 0.934 | 1.18 (0.38–3.66) | 0.77 | 1.28 (0.59–2.76) | 0.534 | 2.88 (0.68–12.10) | 0.149 |
| Exercise at least once per week |  |  |  |  |  |  |  |  |  |  |
| No | Reference |  | Reference |  | Reference |  | Reference |  | Reference |  |
| Yes | 0.90 (0.80–1.00) | 0.052 | 0.76 (0.65–0.89) | 0.001 | 0.97 (0.69–1.37) | 0.863 | 1.02 (0.86–1.22) | 0.808 | 0.68 (0.41–1.14) | 0.145 |
| Medication use^†^ |  |  |  |  |  |  |  |  |  |  |
| Ursodeoxycholic acid | 0.99 (0.73–1.35) | 0.967 | 1.10 (0.74–1.65) | 0.629 | 0.85 (0.41–1.76) | 0.655 | 0.94 (0.48–1.84) | 0.858 | 1.20 (0.42–3.42) | 0.734 |
| Fibrate | 1.04 (0.68–1.59) | 0.862 | 1.06 (0.60–1.85) | 0.842 | 1.33 (0.50–3.54) | 0.566 | 1.24 (0.63–2.44) | 0.541 | 0.57 (0.08–4.17) | 0.577 |
| Glucocorticoid | 1.18 (0.95–1.48) | 0.132 | 1.39 (1.06–1.83) | 0.019 | 1.18 (0.64–2.21) | 0.593 | 0.85 (0.56–1.30) | 0.465 | 1.05 (0.46–2.42) | 0.906 |
| Non-glucocorticoid  immunosuppressive agent^‡^ | 1.18 (0.82–1.69) | 0.383 | 1.51 (0.98–2.31) | 0.06 | 1.00 (0.34–2.93) | 0.995 | 0.68 (0.29–1.61) | 0.381 | 0.97 (0.22–4.32) | 0.973 |

Abbreviations: HR, hazard ratio; CI, confidence interval
^*^ Adjusted for age, sex, socioeconomic status, comorbidities, decompensated cirrhosis, body mass index, smoking, alcohol, exercise, health check-up, and initial medication use
^†^ Medication use was defined as prescription of the relevant drug for ≥30 days within the first 6 months of PBC diagnosis.
^‡^ Non-glucocorticoid immunosuppressive agents include azathioprine, mercaptopurine, and mycophenolate mofetil.

**Supplementary Table 4.** Two-year characteristics of patients with primary biliary cholangitis and matched controls

|  | **PBC** | **Matched controls** | **P-value** |
| --- | --- | --- | --- |
|  | **(n=4,077)** | **(n=17,184)** |  |
| Follow-up duration, median (IQR), y | 6.32 [4.01, 9.60] | 6.61 [4.18, 10.16] | <0.001 |
| Age, mean (SD), y | 56.3 (11.6) | 56.9 (11.7) | 0.004 |
| Age, n (%) |  |  | 0.045 |
| 18–39 years | 312 (7.7) | 1,200 (7.0) |  |
| 40–64 years | 2,759 (67.7) | 11,453 (66.6) |  |
| ≥65 years | 1,006 (24.7) | 4,531 (26.4) |  |
| Female sex, n (%) | 3,360 (82.4) | 14,106 (82.1) | 0.626 |
| Socioeconomic status, n (%) |  |  | <0.001 |
| National health insurance | 3,801 (93.2) | 16,028 (93.3) |  |
| Household income <30% | 783 (19.2) | 3,946 (23.0) |  |
| Household income 30–70% | 1,240 (30.4) | 5,614 (32.7) |  |
| Household income ≥70% | 1,778 (43.6) | 6,468 (37.6) |  |
| Medical aid | 205 (5.0) | 723 (4.2) |  |
| Unknown | 71 (1.7) | 433 (2.5) |  |
| Comorbidities, n (%) |  |  |  |
| Hypertension | 1,328 (32.6) | 5,375 (31.3) | 0.110 |
| Diabetes mellitus | 1,195 (29.3) | 2,998 (17.4) | <0.001 |
| Dyslipidemia | 2,597 (63.7) | 5,168 (30.1) | <0.001 |
| Stroke | 157 (3.9) | 584 (3.4) | 0.157 |
| Chronic kidney disease | 98 (2.4) | 207 (1.2) | <0.001 |
| Osteoporosis | 758 (18.6) | 2,081 (12.1) | <0.001 |
| Anemia | 844 (20.7) | 897 (5.2) | <0.001 |
| Rheumatoid arthritis | 479 (11.7) | 656 (3.8) | <0.001 |
| Hypogonadism | 12 (0.3) | 14 (0.1) | <0.001 |
| CCI score, mean (SD) | 3.1 (2.3) | 1.4 (1.8) | <0.001 |
| CCI score, n (%) |  |  | <0.001 |
| 0 | 220 (5.4) | 7,450 (43.4) |  |
| 1 | 855 (21.0) | 4,132 (24.0) |  |
| 2 | 828 (20.3) | 2,377 (13.8) |  |
| ≥3 | 2,174 (53.3) | 3,225 (18.8) |  |
| Diagnosis period, n (%) |  |  | 0.358 |
| 2007–2010 | 1,087 (26.7) | 4,395 (25.6) |  |
| 2011–2015 | 1,636 (40.1) | 7,017 (40.8) |  |
| 2016–2019 | 1,354 (33.2) | 5,772 (33.6) |  |
| Decompensated cirrhosis, n (%) | 262 (6.4) | 28 (0.2) | <0.001 |
| Long-term medication use^*^, n (%) |  |  |  |
| Ursodeoxycholic acid | 3,764 (92.3) | 206 (1.2) | <0.001 |
| Fibrate | 64 (1.6) | 211 (1.2) | 0.082 |
| Glucocorticoid | 442 (10.8) | 203 (1.2) | <0.001 |
| Non-glucocorticoid immunosuppressive agent^†^ | 329 (8.1) | 19 (0.1) | <0.001 |
| Available for health check-up^‡^ | 3,067 (75.2) | 12,838 (74.7) | 0.493 |
| Body mass index, mean (SD), kg/m^2^ | 23.3 (3.1) | 23.9 (3.3) | <0.001 |
| Body mass index, n (%) |  |  | <0.001 |
| <18.5 kg/m^2^ | 126 (3.1) | 367 (2.1) |  |
| 18.5–22.9 kg/m^2^ | 1,344 (33.0) | 4,915 (28.6) |  |
| 23.0–24.9 kg/m^2^ | 813 (19.9) | 3,211 (18.7) |  |
| ≥25 kg/m^2^ | 784 (19.2) | 4,345 (25.3) |  |
| Unknown | 1,010 (24.8) | 4,346 (25.3) |  |
| Smoking |  |  | 0.902 |
| Never or past | 2,550 (62.5) | 10,682 (62.2) |  |
| Current | 463 (11.4) | 1,969 (11.5) |  |
| Unknown | 1,064 (26.1) | 4,533 (26.4) |  |
| Alcohol consumption |  |  | <0.001 |
| Non-drinker | 2,468 (60.5) | 9,112 (53.0) |  |
| 1–4 times per week | 515 (12.6) | 3,245 (18.9) |  |
| ≥5 times per week | 33 (0.8) | 293 (1.7) |  |
| Unknown | 1,061 (26.0) | 4,534 (26.4) |  |
| Exercise at least once per week |  |  | 0.216 |
| No | 1,494 (36.6) | 6,501 (37.8) |  |
| Yes | 1,516 (37.2) | 6,150 (35.8) |  |
| Unknown | 1,067 (26.2) | 4,533 (26.4) |  |

Abbreviations: CCI, Charlson Comorbidity Index; IQR, interquartile range; PBC, primary biliary cholangitis; SD, standard deviation
^*^ Long-term medication use was defined as prescription of the relevant drug for ≥180 days within the first 2 years of PBC diagnosis.
^†^ Non-glucocorticoid immunosuppressive agents include azathioprine, mercaptopurine, and mycophenolate mofetil.
^‡^ The data for body mass index, smoking, alcohol consumption, and exercise at least once per week were available for participants who underwent health check-up.

**Supplementary Table 5.** Two-year landmark analysis for multivariable Cox regression analysis of overall fragility fracture

|  | **Model 1^*^** |  | **Model 2**^†^ |  | **Model 3**^‡^ |  |
| --- | --- | --- | --- | --- | --- | --- |
|  | HR (95% CI) | P-value | HR (95% CI) | P-value | HR (95% CI) | P-value |
| Primary biliary cholangitis | 1.62 (1.42–1.86) | <0.001 | 1.62 (1.41–1.86) | <0.001 | 1.77 (1.29–2.42) | <0.001 |
| Age | 1.07 (1.06–1.07) | <0.001 | 1.06 (1.06–1.07) | <0.001 | 1.06 (1.06–1.07) | <0.001 |
| Sex |  |  |  |  |  |  |
| Male | Reference |  | Reference |  | Reference |  |
| Female | 2.30 (1.90–2.78) | <0.001 | 2.27 (1.82–2.82) | <0.001 | 2.28 (1.83–2.84) | <0.001 |
| Socioeconomic status |  |  |  |  |  |  |
| Income <30% | Reference |  | Reference |  | Reference |  |
| Income 30–70% | 0.85 (0.68–1.08) | 0.193 | 0.87 (0.68–1.11) | 0.253 | 0.87 (0.68–1.11) | 0.27 |
| Income ≥70% | 0.82 (0.65–1.03) | 0.085 | 0.83 (0.65–1.05) | 0.117 | 0.83 (0.65–1.05) | 0.128 |
| Medical aid | 0.75 (0.60–0.94) | 0.011 | 0.76 (0.60–0.96) | 0.021 | 0.76 (0.60–0.96) | 0.023 |
| Unknown | 0.64 (0.40–1.02) | 0.062 | 0.65 (0.40–1.04) | 0.071 | 0.65 (0.40–1.04) | 0.075 |
| Comorbidity |  |  |  |  |  |  |
| Hypertension | 1.04 (0.91–1.18) | 0.584 | 1.07 (0.90–1.29) | 0.447 | 1.03 (0.90–1.17) | 0.648 |
| Diabetes mellitus | 0.98 (0.85–1.12) | 0.735 | 0.98 (0.81–1.17) | 0.813 | 0.97 (0.85–1.11) | 0.699 |
| Dyslipidemia | 0.97 (0.85–1.10) | 0.613 | 0.89 (0.74–1.06) | 0.185 | 0.97 (0.85–1.10) | 0.614 |
| Stroke | 0.94 (0.73–1.20) | 0.592 | 1.07 (0.79–1.45) | 0.645 | 0.94 (0.73–1.20) | 0.611 |
| Chronic kidney disease | 0.98 (0.65–1.49) | 0.93 | 0.85 (0.48–1.53) | 0.593 | 0.97 (0.64–1.47) | 0.883 |
| Osteoporosis | 1.41 (1.23–1.61) | <0.001 | 1.57 (1.31–1.89) | <0.001 | 1.41 (1.23–1.61) | <0.001 |
| Anemia | 1.15 (0.96–1.38) | 0.136 | 1.17 (0.92–1.49) | 0.212 | 1.14 (0.95–1.37) | 0.16 |
| Rheumatoid arthritis | 1.16 (0.94–1.44) | 0.168 | 1.09 (0.81–1.48) | 0.573 | 1.14 (0.92–1.43) | 0.232 |
| Decompensated cirrhosis | 1.94 (1.38–2.73) | <0.001 | 2.29 (1.52–3.45) | <0.001 | 1.92 (1.36–2.71) | <0.001 |
| Body mass index |  |  |  |  |  |  |
| <18.5 kg/m^2^ |  |  | 1.41 (0.87–2.28) | 0.160 | 1.37 (0.97–1.93) | 0.075 |
| 18.5–22.9 kg/m^2^ |  |  | Reference |  | Reference |  |
| 23.0–24.9 kg/m^2^ |  |  | 1.26 (1.00–1.58) | 0.051 | 0.98 (0.83–1.16) | 0.844 |
| ≥25 kg/m^2^ |  |  | 1.33 (1.06–1.67) | 0.013 | 1.09 (0.93–1.27) | 0.295 |
| Smoking |  |  |  |  |  |  |
| Never or past |  |  | Reference |  | Reference |  |
| Current |  |  | 1.37 (0.97–1.94) | 0.077 | 1.05 (0.80–1.38) | 0.725 |
| Alcohol consumption |  |  |  |  |  |  |
| Non-drinker |  |  | Reference |  | Reference |  |
| 1–4 times per week |  |  | 0.77 (0.56–1.05) | 0.102 | 0.81 (0.65–1.00) | 0.053 |
| ≥5 times per week |  |  | 1.05 (0.49–2.23) | 0.904 | 1.12 (0.64–1.96) | 0.681 |
| Exercise at least once per week |  |  |  |  |  |  |
| No |  |  | Reference |  | Reference |  |
| Yes |  |  | 0.81 (0.67–0.98) | 0.034 | 0.99 (0.87–1.13) | 0.884 |
| Health check-up |  |  | 0.92 (0.72–1.17) | 0.488 | 0.96 (0.81–1.14) | 0.664 |
| Long-term medication use^§^ |  |  |  |  |  |  |
| Ursodeoxycholic acid |  |  |  |  | 0.90 (0.66–1.24) | 0.515 |
| Fibrate |  |  |  |  | 1.11 (0.67–1.84) | 0.686 |
| Glucocorticoid |  |  |  |  | 1.19 (0.88–1.61) | 0.263 |
| Non-glucocorticoid  immunosuppressive agent^∥^ |  |  |  |  | 0.97 (0.62–1.51) | 0.900 |

Abbreviations: HR, hazard ratio; CI, confidence interval
^*^ Model 1: adjusted for age, sex, socioeconomic status, comorbidities, and decompensated cirrhosis
^†^ Model 2: adjusted for age, sex, socioeconomic status, comorbidities, decompensated cirrhosis, body mass index, smoking, alcohol, exercise, and health check-up
^‡^ Model 3: adjusted for age, sex, socioeconomic status, comorbidities, decompensated cirrhosis, body mass index, smoking, alcohol, exercise, health check-up, and long-term medication use
^§^ Long-term medication use was defined as prescription of the relevant drug for ≥180 days within the first 2 years of PBC diagnosis.
^∥^ Non-glucocorticoid immunosuppressive agents include azathioprine, mercaptopurine, and mycophenolate mofetil.

**Supplementary Table 6.** Two-year landmark analysis for multivariable Cox regression analysis of site-specific fragility fractures

|  | **Overall fragility fracture** | | **Vertebral Fracture** | | **Hip fracture** | | **Distal radius fracture** | | **Proximal humerus fracture** | |
| --- | --- | --- | --- | --- | --- | --- | --- | --- | --- | --- |
|  | HR^*^ (95% CI) | P-value | HR^*^ (95% CI) | P-value | HR^*^ (95% CI) | P-value | HR^*^ (95% CI) | P-value | HR^*^ (95% CI) | P-value |
| Primary biliary cholangitis | 1.77 (1.29–2.42) | <0.001 | 1.82 (1.18–2.81) | 0.006 | 1.84 (0.88–3.81) | 0.103 | 1.70 (0.97–2.96) | 0.063 | 0.91 (0.24–3.48) | 0.892 |
| Age | 1.06 (1.06–1.07) | <0.001 | 1.08 (1.07–1.09) | <0.001 | 1.10 (1.08–1.12) | <0.001 | 1.03 (1.02–1.04) | <0.001 | 1.04 (1.02–1.07) | <0.001 |
| Sex |  |  |  |  |  |  |  |  |  |  |
| Male | Reference |  | Reference |  | Reference |  | Reference |  | Reference |  |
| Female | 2.28 (1.83–2.84) | <0.001 | 2.02 (1.52–2.69) | <0.001 | 1.89 (1.15–3.10) | 0.01 | 3.13 (2.01–4.87) | <0.001 | 3.13 (1.25–7.80) | 0.014 |
| Socioeconomic status |  |  |  |  |  |  |  |  |  |  |
| Income <30% | Reference |  | Reference |  | Reference |  | Reference |  | Reference |  |
| Income 30–70% | 0.87 (0.68–1.11) | 0.27 | 0.72 (0.52–0.99) | 0.044 | 0.57 (0.32–1.04) | 0.069 | 1.16 (0.72–1.87) | 0.533 |  |  |
| Income ≥70% | 0.83 (0.65–1.05) | 0.128 | 0.77 (0.57–1.04) | 0.086 | 0.76 (0.44–1.31) | 0.323 | 0.99 (0.62–1.57) | 0.97 |  |  |
| Medical aid | 0.76 (0.60–0.96) | 0.023 | 0.67 (0.50–0.90) | 0.007 | 0.80 (0.49–1.31) | 0.377 | 0.93 (0.59–1.47) | 0.756 |  |  |
| Unknown | 0.65 (0.40–1.04) | 0.075 | 0.77 (0.43–1.40) | 0.396 | 0.80 (0.24–2.69) | 0.723 | 0.37 (0.13–1.08) | 0.068 |  |  |
| Comorbidity |  |  |  |  |  |  |  |  |  |  |
| Hypertension | 1.03 (0.90–1.17) | 0.648 | 1.07 (0.89–1.28) | 0.469 | 1.24 (0.85–1.82) | 0.268 | 0.89 (0.71–1.12) | 0.328 |  |  |
| Diabetes mellitus | 0.97 (0.85–1.11) | 0.699 | 0.98 (0.81–1.17) | 0.805 | 1.17 (0.82–1.68) | 0.39 | 0.83 (0.65–1.07) | 0.159 |  |  |
| Dyslipidemia | 0.97 (0.85–1.10) | 0.614 | 0.88 (0.73–1.05) | 0.155 | 1.22 (0.85–1.74) | 0.288 | 0.99 (0.80–1.23) | 0.941 | 1.84 (1.15–2.94) | 0.011 |
| Stroke | 0.94 (0.73–1.20) | 0.611 | 1.08 (0.80–1.46) | 0.617 | 1.04 (0.61–1.79) | 0.878 | 0.65 (0.37–1.14) | 0.13 |  |  |
| Chronic kidney disease | 0.97 (0.64–1.47) | 0.883 | 0.83 (0.46–1.49) | 0.537 | 2.54 (1.29–5.02) | 0.007 | 0.68 (0.25–1.88) | 0.462 |  |  |
| Osteoporosis | 1.41 (1.23–1.61) | <0.001 | 1.55 (1.29–1.86) | <0.001 | 0.94 (0.65–1.37) | 0.746 | 1.44 (1.14–1.81) | 0.003 |  |  |
| Anemia | 1.14 (0.95–1.37) | 0.16 | 1.15 (0.90–1.47) | 0.258 | 1.33 (0.86–2.08) | 0.202 | 1.11 (0.80–1.56) | 0.536 |  |  |
| Rheumatoid arthritis | 1.14 (0.92–1.43) | 0.232 | 1.04 (0.76–1.42) | 0.807 | 0.94 (0.48–1.82) | 0.842 | 1.12 (0.77–1.63) | 0.539 |  |  |
| Decompensated cirrhosis | 1.92 (1.36–2.71) | <0.001 | 2.17 (1.44–3.30) | <0.001 | 1.66 (0.67–4.14) | 0.276 | 1.07 (0.47–2.47) | 0.867 |  |  |
| Health check-up | 0.96 (0.81–1.14) | 0.664 | 0.92 (0.72–1.18) | 0.505 | 0.52 (0.31–0.85) | 0.01 | 1.29 (0.98–1.70) | 0.07 | 0.57 (0.31–1.04) | 0.068 |
| Body mass index |  |  |  |  |  |  |  |  |  |  |
| 18.5–22.9 kg/m^2^ | Reference |  | Reference |  | Reference |  | Reference |  | Reference |  |
| <18.5 kg/m^2^ | 1.37 (0.97–1.93) | 0.075 | 1.41 (0.87–2.28) | 0.16 | 3.88 (1.85–8.13) | <0.001 | 0.60 (0.28–1.25) | 0.171 | 4.76 (1.82–12.44) | 0.001 |
| 23.0–24.9 kg/m^2^ | 0.98 (0.83–1.16) | 0.844 | 1.25 (1.00–1.58) | 0.054 | 1.04 (0.59–1.83) | 0.889 | 0.74 (0.57–0.98) | 0.036 | 0.67 (0.30–1.50) | 0.327 |
| ≥25 kg/m^2^ | 1.09 (0.93–1.27) | 0.295 | 1.33 (1.06–1.66) | 0.013 | 1.38 (0.84–2.27) | 0.206 | 0.94 (0.73–1.20) | 0.594 | 1.23 (0.63–2.37) | 0.546 |
| Smoking |  |  |  |  |  |  |  |  |  |  |
| Never or past | Reference |  | Reference |  | Reference |  | Reference |  | Reference |  |
| Current | 1.05 (0.80–1.38) | 0.725 | 1.37 (0.96–1.94) | 0.08 | 1.37 (0.71–2.67) | 0.349 | 0.73 (0.43–1.24) | 0.241 |  |  |
| Alcohol consumption |  |  |  |  |  |  |  |  |  |  |
| Non-drinker | Reference |  | Reference |  | Reference |  | Reference |  | Reference |  |
| 1–4 times per week | 0.81 (0.65–1.00) | 0.053 | 0.77 (0.56–1.06) | 0.106 | 0.54 (0.23–1.24) | 0.146 | 0.84 (0.61–1.17) | 0.304 |  |  |
| ≥5 times per week | 1.12 (0.64–1.96) | 0.681 | 1.05 (0.49–2.24) | 0.897 | 1.73 (0.54–5.54) | 0.355 | 1.33 (0.54–3.24) | 0.535 |  |  |
| Exercise at least once per week |  |  |  |  |  |  |  |  |  |  |
| No | Reference |  | Reference |  | Reference |  | Reference |  | Reference |  |
| Yes | 0.99 (0.87–1.13) | 0.884 | 0.81 (0.67–0.99) | 0.036 | 1.14 (0.76–1.71) | 0.538 | 1.13 (0.91–1.40) | 0.257 |  |  |
| Long-term medication use^†^ |  |  |  |  |  |  |  |  |  |  |
| Ursodeoxycholic acid | 0.90 (0.66–1.24) | 0.515 | 1.09 (0.71–1.69) | 0.682 | 0.99 (0.47–2.10) | 0.989 | 0.70 (0.40–1.23) | 0.218 | 1.57 (0.42–5.93) | 0.502 |
| Fibrate | 1.11 (0.67–1.84) | 0.686 | 1.18 (0.62–2.27) | 0.610 | 1.06 (0.30–3.71) | 0.927 | 1.33 (0.58–3.08) | 0.501 |  |  |
| Glucocorticoid | 1.19 (0.88–1.61) | 0.263 | 1.62 (1.11–2.36) | 0.012 | 0.84 (0.36–2.01) | 0.704 | 0.88 (0.51–1.51) | 0.630 | 0.72 (0.25–2.05) | 0.540 |
| Non-glucocorticoid  immunosuppressive agent^‡^ | 0.97 (0.62–1.51) | 0.900 | 0.97 (0.55–1.70) | 0.908 | 1.92 (0.68–5.42) | 0.218 | 1.07 (0.50–2.31) | 0.862 | 1.69 (0.47–6.13) | 0.423 |

Abbreviations: HR, hazard ratio; CI, confidence interval
^*^ Adjusted for age, sex, socioeconomic status, comorbidities, decompensated cirrhosis, body mass index, smoking, alcohol, exercise, health check-up, and long-term medication use
^†^ Long-term medication use was defined as prescription of the relevant drug for >180 days within the first 2 years of PBC diagnosis.
^‡^ Non-glucocorticoid immunosuppressive agents include azathioprine, mercaptopurine, and mycophenolate mofetil.

**Supplementary Table 7.** Risk for overall fragility fractures for patients with PBC, compared with their matched controls

|  | **HR* (95% CI)** | **P-value** | **P-value for Interaction** |
| --- | --- | --- | --- |
| **Sex (n=24,744)** |  |  | 0.006 |
| Male | 2.53 (1.84 - 3.48) | <0.001 |  |
| Female | 1.59 (1.41 - 1.79) | <.0001 |  |
| **Menopause (n=20,295)** |  |  | 0.309 |
| Woman aged≤ 50 | 1.89 (1.34 - 2.66) | <0.001 |  |
| Woman aged > 50 | 1.57 (1.38 - 1.78) | <.0001 |  |

Abbreviations: HR, hazard ratio; CI, confidence interval
* Adjusted for age, income, comorbidities, BMI, smoking, drinking, and physical activity

**Supplementary Table 8.** Overall incidence of fragility fractures stratified by sex and menopausal status

|  | **Male (N=4,449)** | | | **Female (N=20,295)** | | |
| --- | --- | --- | --- | --- | --- | --- |
|  | **Observed**  **cases** | **Sum  of PYs** | **Incidence rate^*^ (95% CI)** | **Observed cases** | **Sum  of PYs** | **Incidence rate^*^ (95% CI)** |
| Overall fracture | 167 | 26,513.9 | 6.3 (5.4–7.3) | 1,712 | 125,037.4 | 13.7 (13.1–14.4) |
| Vertebral fracture | 105 | 26,748.4 | 3.9 (3.2–4.7) | 912 | 128.722.7 | 7.1  (6.6–7.6) |
| Hip fracture | 31 | 27,100.6 | 1.1  (0.8–1.6) | 213 | 131,768.9 | 1.6  (1.4–1.8) |
| Distal radius fracture | 34 | 27,073.9 | 1.3  (0.9–1.8) | 649 | 129,491.4 | 5.0  (4.6–5.4) |
| Proximal humerus fracture | 8 | 27,177.4 | 0.3  (0.1–0.6) | 110 | 132,116.5 | 0.8  (0.7–1.0) |
|  | **Premenopausal women (N=5,732)** | | | **Postmenopausal women (14,563)** | | |
|  | **Observed**  **cases** | **Sum  of PYs** | **Observed**  **cases** | **Sum  of PYs** | **Observed**  **cases** | **Sum  of PYs** |
| Overall fracture | 145 | 39551.6 | 3.7 (3.1–4.3) | 1,567 | 85485.8 | 18.3 (17.4–19.3) |
| Vertebral fracture | 47 | 40022.2 | 1.2 (0.9–1.6) | 865 | 88700.5 | 9.8 (9.1–10.4) |
| Hip fracture | 5 | 40205.0 | 0.1 (0.0–0.3) | 208 | 91563.9 | 2.3  (2.0–2.6) |
| Distal radius fracture | 84 | 39783.6 | 2.1 (1.7–2.6) | 565 | 89707.8 | 6.3 (5.8–6.8) |
| Proximal humerus fracture | 16 | 40169.0 | 0.4 (0.2–0.6) | 94 | 91947.5 | 1.0 (0.8–1.3) |

Abbreviations: CI, confidence interval; PBC, primary biliary cholangitis; PY, person-years
^*^ Incidence rate per 1,000 PYs.
